# Handling and standardization of EBUS needle aspiration in NSCLC patients: The value of the cell block, a monoinstitutional experience

**DOI:** 10.1111/1759-7714.14581

**Published:** 2022-07-22

**Authors:** Paola Parente, Cristiano Carbonelli, Giovanni Biancofiore, Andi Sukthi, Concetta Martina Di Micco, Matteo Vairo, Paolo Fuso, Marco Taurchini, Paolo Graziano

**Affiliations:** ^1^ Pathology Unit Fondazione IRCCS Casa Sollievo della Sofferenza San Giovanni Rotondo Italy; ^2^ Pneumology Unit Department of Medical Sciences, Fondazione IRCCS Casa Sollievo della Sofferenza San Giovanni Rotondo Italy; ^3^ Oncology Unit Department of Medical Sciences, Fondazione IRCCS Casa Sollievo della Sofferenza San Giovanni Rotondo Italy; ^4^ Department of Medical and Surgical Sciences, Institute of Respiratory Disease Policlinico Universitario ‘Riuniti’ di Foggia, University of Foggia Foggia Italy; ^5^ Thoracic Surgery Unit Fondazione IRCCS Casa Sollievo della Sofferenza San Giovanni Rotondo Italy

**Keywords:** cell block, cytopathology, EBUS TBNA, lung cancer diagnosis, non‐small cell lung cancer

## Abstract

**Background:**

Lung cancer is the main cause of cancer‐related death worldwide, and 85% of all lung tumors are non‐small cell lung cancers (NSCLC). More than 60% of all lung tumors are diagnosed at an advanced stage, leading to poor prognosis. Given the growing demand for NSCLC profiling for selection of the most appropriate therapy, the acquisition of adequate tumor samples has become increasingly crucial, mostly in advanced NSCLC patients due to old age and/or comorbidities. Being a mini‐invasive sampling technique, endobronchial ultrasound‐guided transbronchial needle aspiration (EBUS‐TBNA) represents a valuable alternative to traditional transthoracic or surgical sampling in these patients, and perfoming cell block (CB) could be crucial to maximize the potential biological information. The aim of this study is to describe a monoinstitutional interprofessional experience in handling EBUS‐TBNA and CB in 464 patients.

**METHODS:**

We retrospectively collected all the consecutive CBs obtained from EBUS TBNA performed between 2014 and 2021 on the lung lesions or mediastinal lymph nodes. All the CBs were handled in a standardized method.

**RESULTS:**

A total of 95.5% (448/464 samples) of adequacy for site and 92.6% (430/464) of adequacy for diagnosis were observed. Moreover, in the adenocarcinoma histotype, ALK, ROS1 and tumor proportion score (TPS) PD‐L1 assessment by IHC was possible in 96% (140/146) of cases, and molecular profile was obtained in 93.8% (137/146) of cases. In the squamous cell carcinoma histotype, TPS PD‐L1 assessment was possible in 81% (13/16) of cases. All four CB results obtained from carcinoma NOS were adequate for ALK, ROS1 and PD‐L1 assessment and molecular profiling. All 39 metastatic samples from extra‐pulmonary primary were adequate for immunohistochemical characterization and molecular profiling. Finally, reporting of the tumor sample adequacy to the clinicians took a median time of about 30 h (range: 24–80 h).

**Conclusion:**

Careful cytological smear management together with the handling and standardization of CB obtained from EBUS‐TBNA could represent an effective method to increase the adequacy of the tumor specimen for both diagnosis and molecular profile.

## Graphical abstract

In advanced NSCLC patients, a less invasive procedure such as EBUS‐TBNA allows a more accurate acquisition of samples. Careful cytological smear management together with the handling and standardization of CB obtained from EBUS‐TBNA could represent an effective method to increase the adequacy of the tumor specimen for both diagnosis and molecular profile.

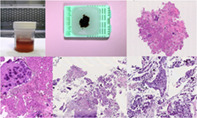

## INTRODUCTION

Lung cancer is the main cause of cancer‐related death worldwide. Non‐small cell lung cancer (NSCLC) accounts for approximately 85% of all histotypes.^1^ Sixty per cent of NSCLC patients are usually diagnosed with advanced disease not suitable for surgical treatment,^2^ and molecular tumor profiling has become essential to select the most appropriate therapy.^1^, ^3^, ^4^ In particular, 10%–15% of NSCLCs show epidermal growth factor receptor (EGFR) mutations, suggesting the use of specific tyrosine kinase inhibitors (TKI) such as gefitinib, erlotinib, afatinib and osimertinib.^5^ Rearrangement of the anaplastic lymphoma kinase (ALK) gene is observed in roughly 5% of NSCLCs, predicting response to ALK inhibitors such as crizotinib, ceritinib, alectinib, and brigatinib.^6^ It has previously been reported that 1%–2% of NSCLCs are characterized by ROS proto‐oncogene 1 (ROS1) rearrangement, which is predictive of response to crizotinib.^7^ Kirsten rat sarcoma (KRAS) mutations, mainly detected in smokers affected by adenocarcinoma, associated with brain metastasis and worse prognosis,^8^ were acknowledged as undruggable until recent potential effective inhibition.^9^ V600E point mutation of the v‐raf murine sarcoma viral oncogene homolog B1 (BRAF) gene in 1%–5% of NSCLCs has also emerged as a valuable therapeutic target.^10^

Finally, the programmed death‐ligand 1 (PD‐L1) assessment evaluated by immunohistochemistry (IHC) as tumor proportion score (TPS), is one of the required indicators to select NSCLC patients for immune checkpoint inhibitor therapy.^11^ Patients with advanced NSCLC and PD‐L1 TPS > 50% benefit from first‐line treatment based on the anti‐PD‐1 agent pembrolizumab, obtaining improved survival and reduced adverse effects compared to standard chemotherapy.^12^ Pembrolizumab and platinum‐based chemotherapy are indicated as first‐line treatment in patients with PD‐L1 TPS of 1%–49%. Pembrolizumab can also be administered as second‐line treatment in the presence of PD‐L1 TPS >1%.^13^, ^14^

The most recent guidelines from the College of American Pathologists (CAP), the International Association for the Study of Lung Cancer (IASLC) and the Association for Molecular Pathology (AMP) recommend molecular testing at the time of initial diagnosis in patients with advanced NSCLC, regardless of clinical characteristics (Reflex test).^15^ The current NCCN guidelines have expanded the indication for EGFR and ALK testing to nonsquamous NSCLC and NSCLC not otherwise specified (NOS) as well as to nonsmokers with metastatic squamous cell carcinoma histotype, and recommend PD‐L1 TPS assessment at the initial diagnosis of advanced NSCLC.^11^

To achieve a complete diagnosis, including histotype and molecular and immunological profiling, it is imperative to acquire an adequate tumor sample.^16^ Unfortunately, in advanced NSCLC patients, collecting sufficient tumor specimens can be challenging due to old age and/or comorbidities.^2^ Recently, minimally invasive procedures such as endobronchial ultrasound‐guided transbronchial needle aspiration (EBUS‐TBNA) have enabled the acquisition of tumor samples mainly as cytological material.^16^ Echoendoscopic sampling should be the primary approach to achieve a diagnosis and stage the lung cancer as it is a less invasive procedure and has fewer complications compared to the traditional transthoracic or surgical sampling techniques.^17^ Of note, a survival benefit has been demonstrated in patients whose diagnostic and staging assessment was made by EBUS‐TBNA.^18^ In this setting, the diagnostic yield of cytological aspiration through endoscopic techniques depends on the availability of the echoendoscopic guidance to mediastinal and pulmonary targets, as this technology improves diagnostic adequacy and achievement of molecular profiling.^19^

The most widespread method to analyze cytological samples from EBUS‐TBNA is to smear samples on slides. However, suboptimal handling, fixation, and staining techniques can cause cell overlapping or overcrowding, cell loss, artifacts, and poor background staining.^20^ Moreover, smearing does not allow minimal immunohistochemical analysis to be performed easily for the definition of the histotype of poorly differentiated cases.^21^ On the other hand, alcohol‐based cytology fixatives allow a better preservation of the nucleic acids that will be used to perform molecular analysis.^22^

The cell block (CB) technique is one of the oldest methods for the evaluation of body cavity fluids and a well‐established tool for diagnosis and also useful for molecular profiling of pulmonary neoplasms.^21^, ^23^ A proper CB setting allows the preservation of cellularity, architecture and details of the nucleus and cytoplasm adequately and satisfactorily; moreover, IHC and molecular analyses can also be performed.^20^ In fact, architectural patterns such as glands, sheets, three‐dimensional cell clusters, and cell balls are commonly displayed on CB leading, moreover, to a confidential IHC assessment.^20^, ^21^ Finally, CB specimens can be comfortably collected, whereas smear storage remains a debated issue.^23^

On the other hand, formalin fixation required for CB setting results in significant degradation of macromolecules (DNA, RNA, and proteins), decreasing next‐generation sequencing (NGS) feasibility.^22^

In advanced NSCLC patients, using smears as a main source for molecular testing and CB for histotype definition and for ALK, ROS1 and PD‐L1 assessment may represent a wise way of handling EBUS neoplastic specimens.^24^

In the current literature, it is reported that CB preparation from EBUS‐TBNA samples has proved to be able to increase the diagnostic yield by 7% and to provide material for genetic analysis in 60% of the patients with metastatic NSCLC.^25^ Moreover, there is only limited data, all from small case series, on the utility of CB from EBUS‐TBNA samples.^26^

In this study on a consecutive series of 464 EBUS samples from pulmonary masses and mediastinal lymph nodes, we aim to describe: (1) CB adequacy for site assessment in terms of representativeness of lung or lymph node tissue, respectively; (2) CB adequacy for diagnosis in terms of availability of sufficient tissue to achieve a pathological diagnosis and (3) the adequacy of CB in terms of neoplastic cellularity for molecular analyses and other bioprognostic factors in advanced NSCLC patients.

## METHODS

All consecutive EBUS‐TBNAs performed between 2014 and 2021 on suspected primary lung or metastatic to lung cancer patients with/without mediastinal lymph node involvement and mediastinal lymph adenopathy of unknown etiology were included in this study. Patient demographics, anatomic site biopsied, procedure details, and stage of disease were collected.

All information regarding human material was anonymized, and all samples were handled in compliance with the Declaration of Helsinki (https://www.wma.net/what-we-do/medical-ethics/declaration-of-helsinki/).

### EBUS‐TBNA procedure

The EBUS‐TBNA procedure was always planned by the interventional pulmonologist after the re‐evaluation of radiological imaging to establish the most adequate site of sampling. In the case of metastatic pulmonary disease, the easiest site of sampling was preferred (pulmonary lesion/mass or lymph nodes); otherwise, in cases of neoplasm restricted to the lung, parenchymal sampling was considered.

EBUS‐TBNA was performed using an ultrasound bronchoscope (BF‐UC180F; Olympus) in combination with an ultrasound processor (EU‐ME2; Olympus) under local anesthesia and conscious sedation. The ultrasound bronchoscope was passed through the mouth to the trachea, then the ultrasound transducer was conducted towards endoscopic anatomical landmarks in search of lesions close to the airway wall. Once target lesions were visualized by ultrasound imaging, needle aspirations with a 22G needle were performed under real‐time ultrasound guidance (NA‐201SX‐4022; Olympus). The aspirated specimen was promptly pushed out by a needle stylet into a container filled with a 10% neutral buffered formalin solution. EBUS‐TBNA was performed with at least four passes for each target. No smears for rapid on site evaluation (ROSE) were performed.

No complications after EBUS‐TBNA procedures were reported.

### CB procedure

**FIGURE 1:**
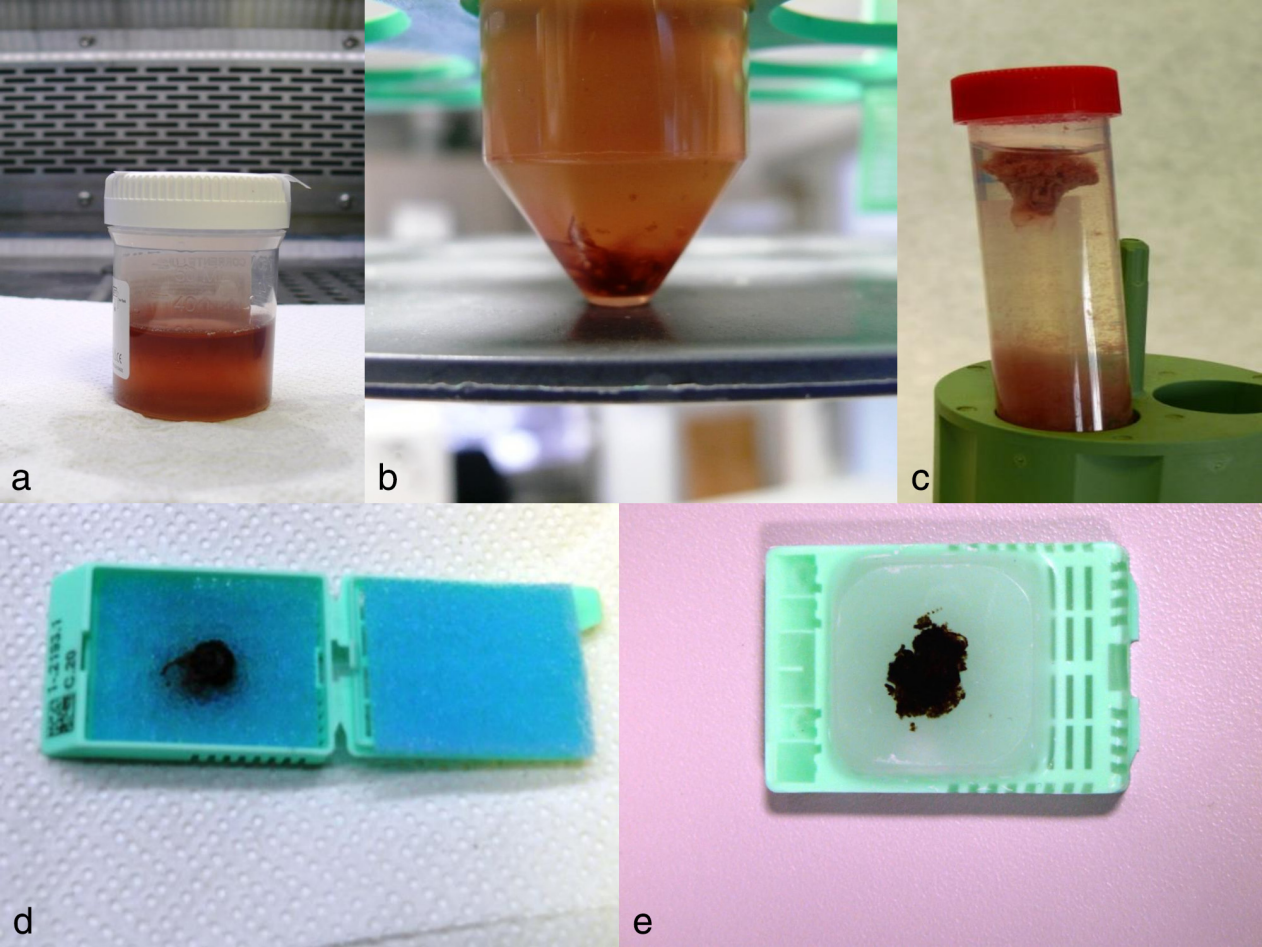
Cytological specimens were placed into a container with 10% neutral buffered formalin solution (a,b); solid clot (c) into cell for processing (d) and embedded in paraffin (e)

**FIGURE 2:**
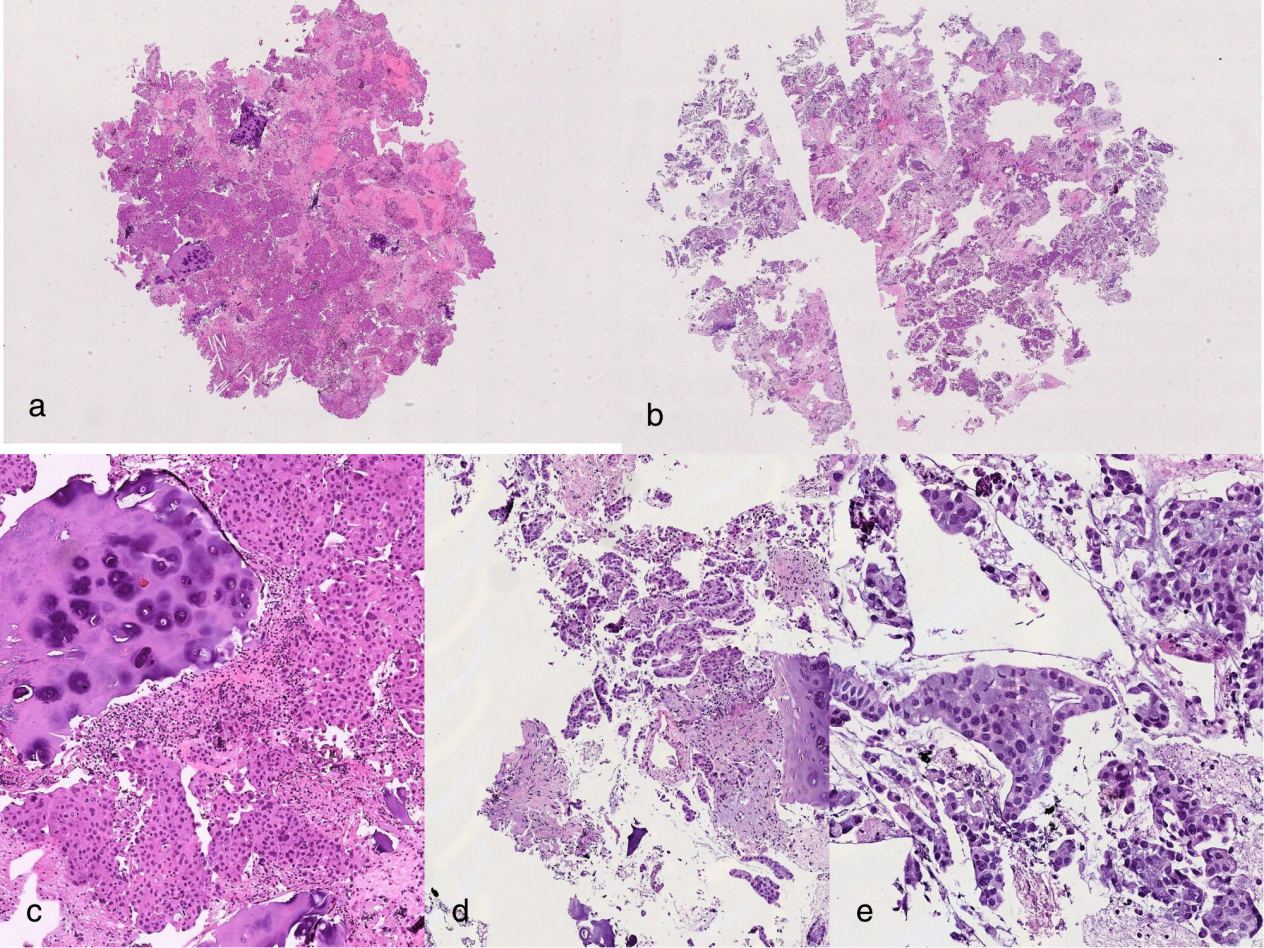
Haematoxilin and eosin (H&E) stained sections (4×) from mediastinal lymph node (a) and pulmonary mass (b), respectively. (c) Cartilage and lymphocytes adjacent to neoplastic component (H&E, 10×). (d) Adenocarcinoma histotype is visible on H&E stained section (10×; E, 20×)

Cytological samples obtained from EBUS‐TBNA were handled as previously described.^20^ Briefly, samples were fixed in a 10% neutral buffered formalin solution and automatically processed and embedded in paraffin (Figure 1). Then, 3μm‐thick sections were stained with hematoxylin and eosin (H&E) and analyzed for adequacy and diagnosis (Figure 2).

To avoid preanalytic artifacts that could affect morphological interpretation, IHC, FISH and molecular analyses, an internal agreement among pathologists, technicians and interventional pneumologists was made on the timing of sample collections and delivery to the Pathology Unit. All the TBNAs were performed in the morning and delivered to the Pathology laboratory in the early afternoon, at the latest; the samples were processed the same day with a time‐fixation range of 6–12 h, avoiding hyperfixation artifacts. From March 2014, our internal CB preparation protocol was improved with the help of an expert cytology technician (GB).^20^

An internal quality control of the cytological activity, to ensure that tumor classification would be made according to the WHO Classification of Lung Tumors^27^ and material spared for prognostic and/or predictive purposes, was performed by at least two pathologists or one pathologist and one cytologist.

A cytological sample of the lymph nodes was defined adequate for site if any of the following indicators was satisfied: germinal center fragments or at least 100 lymphocytes per field (evaluated in at least five fields at × 100 magnification). Moreover, a cytological sample was defined as adequate for diagnosis if it led to a definitive diagnosis of malignancy (primary or metastatic) or of necrotizing/non‐necrotizing granuloma in the appropriate clinical context. Finally, a specimen enclosing a range of 100–400 viable neoplastic cells was termed “adequate for molecular assessment”.^16^, ^26^

In all patients eligible for target therapy as established by the Italian Medicines Agency (AIFA), molecular analysis was performed on the most adequate technology and platform (NGS, pyrosequencing method, fluorescence in situ hybridization) according to the amount and the quality of tumor sample.

## RESULTS

Between 2014 and 2021, a total of 464 patients were enrolled at the Research Hospital “Fondazione IRCCS Casa Sollievo della Sofferenza” in San Giovanni Rotondo, Foggia, Italy; the median age was 67 years (range 17–88), and male to female ratio was 2.7/1 (339 males, 125 females). In 351 cases (75.6%), a single mediastinal lymph node (N1 or N2) sample was performed. In 109 cases (23.5%), a double mediastinal lymph node sampling was obtained. In particular, 62 N10 stations, 118 N11 stations, 162 N4 stations and 222 N7 stations were sampled, respectively. Four pulmonary lesions (0.9%) were obtained (Table 1).

**TABLE 1:** Demographic and anatomical findings

Characteristic	N 464	%
Gender		
Male	339	73%
Female	125	27%
Age		
Mean age, years (range)	67 years (range 17–88)	
Site		
Monostation N1/N2 lymph node	351	75.6%
Multiple N2/N1 + N2 lymph nodes	109	23.5%
Lung lesion or mass	4	0.9%

Of 464 cases, 448 (95.5%) and 430 (92.6%) were adequate for site and diagnosis, respectively. Of the 430 samples that were found adequate for diagnosis, 238 (55%) resulted to be primary lung cancers; mesothelioma was diagnosed in just one patient (0.2%); pulmonary metastasis was shown in 39 cases (9%); lymphoid tissue without disease (neoplastic and non‐neoplastic) was observed in 104 patients (24%); non‐necrotizing and necrotizing granulomas were detected in 15 (3.3%) and 29 patients (8%), respectively (Table 2).

**TABLE 2:** Adequacy for site and for diagnosis in CB with respective final cytological diagnosis

Total CB	464	
Adequate for site	448	95.5%
Adequate for diagnosis	430	92.6%
Primitive lung cancer	238	55%
Adenocarcinoma	157	66%
Squamous cell carcinoma	33	13.8%
SCLC	35	14.7%
Carcinoma, NOS	4	2%
Lymphoma (Hodgkin's lymphoma)	3	1.35%
Carcinoid	3	1.35%
Combined NSCLC + SCLC	2	0.8%
Mesothelioma	1	0.2%
Metastasis to lung	39	9%
Lower GI (colon‐rectum)	5	12.8%
Breast	12	30.7%
Kidney/bladder/prostate	10	25.6%
Head and neck	1	3.3%
Melanoma	1	3.3%
Upper GI (esophagus/stomach/pancreas)	3	7.8%
Sarcoma	1	3.3%
Cordoma	1	3.3%
Female genital tract	4	9.9%
Lymphoid tissue	104	24%
Inflammatory/necrotizing granulomas	15	3.3%
Non‐necrotizing granuloma (suggestive of sarcoidosis) Abbreviations: CB, cell block; GI, gastrointestinal; NOS, not otherwise specified; NSCLC, non‐small cell lung cancer; SCLC, small cell lung cancer	29	8%

Of the 238 primary lung cancers, 156 (66%) were adenocarcinomas, 33 (13.8%) squamous cell carcinomas and 35 (14.7%) small cell carcinomas (SCLC).

Moreover, a diagnosis of carcinoma NOS was established in four cases (2%), Hodgkin's lymphoma in three patients (1.35%) and carcinoid in the other three patients (1.35%).

Finally, a combined NSCLC + SCLC diagnosis was also suggested in two cases (0.8%).

In 39 patients with pulmonary metastases from an extra‐thoracic primary, five cases (12.8%) were from the lower gastrointestinal tract, 12 cases (30.7%) from the breast, 10 cases (25.6%) from the urinary system and prostate, one case (3.3%) from the head and neck district, four cases (9.9%) from the female genital tract, and three cases (7,8%) from the upper gastrointestinal tract.

Finally, metastasis from melanoma (3.3%), sarcoma (3.3%) and chordoma (3.3%) were observed in three distinct patients, respectively.

In 123 out of 231 patients with primary pulmonary carcinoma, IHC analyses were necessary to assign the specific histotype according to the WHO classification (Table 3).

**TABLE 3:** IHC in primary lung carcinoma; ALK, ROS1, PD‐L1 assessment and molecular profiling in advanced NSCLC CB with respective final cytological diagnosis

NSCLC + SCLC patients	245	
IHC tests done	133	
Advanced stage NSCLC (IIIb + IV)	164/196	83.6%
Advanced NSCLC, Adenocarcinoma type	146	96%
Adequate for ALK, ROS, PD‐L1 IHC profiling	140	96%
Inadequate for ALK, ROS, PD‐L1 IHC profiling	6	4%
Adequate for molecular profiling	137	93.8%
Indequate for molecular profiling	9	6.2%
Advanced NSCLC, squamous cell type	16	
Adequate for PDL1 IHC profiling	13	81%
Advanced NSCLC, NOS type	2	
Adequate for ALK, ROS, PD‐L1 IHC profiling	2	100%
Adequate for molecular profiling	2	100%

Abbreviations: ALK, anaplastic lymphoma kinase; IHC, immunohistochemistry; NOS, not otherwise specified; NSCLC, non‐small cell lung cancer; PD‐L1, programmed cell‐death ligand 1; ROS1, ROS proto‐oncogene 1; SCLC, small cell lung cancer.

Stage IIIb–IV characterized 164 (83.6%) NSCLC patients, and 146 patients were affected by adenocarcinoma. In total, 140 out of 146 samples (96%) of adenocarcinoma were adequate for ALK, ROS1 and TPS PD‐L1 immunohistochemical assessment, and 137/146 (93.8%) were adequate for molecular profiling. Sixteen patients were diagnosed with squamous cell carcinoma, and 13/16 samples (81%) were adequate for TPS PD‐L1 assessment. Two patients received a diagnosis of carcinoma NOS, and all samples were adequate for ALK, ROS1 and TPS PD‐L1 immunohistochemical assessment and molecular profiling (Table 3).

All 39 cases with pulmonary metastases (100%) were adequate for either IHC characterization or molecular analysis, in terms of neoplastic cellularity. However, we only performed molecular profiling in metastasis from lower gastrointestinal tract (5 cases) and from melanoma (1 case), based on current AIFA guidelines.

The Hodgkin's lymphoma cases were also characterized by IHC, as previously described.^20^, ^28^

Finally, reporting of the tumor sample adequacy to the clinicians took a median time of about 30 h (range: 24–80 h).

## DISCUSSION

Lung cancer is the most common malignancy worldwide and remains the first cause of cancer death in both men and women. Around 85% of all lung cancers are NSCLC, and 60% of all lung tumors are diagnosed at an advanced stage with a median five‐year survival of 15%.^29^ In both resectable and nonresectable patients, a pathological confirmation of malignancy and subtyping is needed. Moreover, acquiring a tumor sample from NSCLC advanced patients to perform molecular studies can be challenging.^3^ In 30%–40% of advanced NSCLC patients, only cytology specimens acquired by EBUS‐TBNA are usually available for diagnosis and molecular profiling.^30^ Although current guidelines by interventional pulmonologists do not recommend diagnostic superiority of CBs over smears,^31^, ^32^ preservation of morphological details may render CB useful either for immunohistochemical and also molecular analyses.^20^

CB preparation has been reported to reduce cellular dispersal, increase carcinoma pattern recognition and the possibility of obtaining multiple sections for routine, histochemical and IHC stainings and molecular analysis.^21^, ^23^ The well‐recognized limitations of CB (relatively low DNA yield, detrimental impact of formalin fixation, and time‐consuming specimen processing) make smears preferable for NGS testing and CB for ALK, ROS1 and PD‐L1 assessment and FISH assays.^33^, ^34^

Until now, few data on CB comprehensive adequacy for site, diagnosis and predictive and prognostic profiling in EBUS‐TBNA case series are available.

In this study on a large monocentric series of EBUS‐TBNA procedures, we describe CB adequacy for site, diagnosis and predictive and prognostic profiling in a retrospective series standardized for sampling method (both endoscopic procedure and pulmonologist's expertise), time‐fixation (no more than 12 h), and handling of specimens (fixative solution and clot preparation).

In our Institute, EBUS‐TBNA procedures were performed by the interventional pulmonologists with specific experience in the use of the broncoendoscopic procedure allowing sampling under real‐time ultrasound guidance. All samples were acquired with a 22 G needle with no less than four passes for each target.

To date, the International Association for the Study of Lung Cancer (IASLC) has specified the optimal time for fixation for biopsy (6–48 h) and surgical specimens (24–48 h) but not for cytological material.^35^ Similarly, the Guidelines For Clinical Laboratories by the Canadian Association of Pathologists—Association Canadienne Des Pathologistes (CAP‐ACP) recommend handling CBs with the preanalytical procedures applied to histological samples.^36^ A recognized preanalytical factor for IHC is the length of fixation time; obviously, the IHC results in CBs could be affected by a prolonged and/or improper fixative solution and/or fixation time.

In our Institute, cytological samples obtained through EBUS‐TBNA are fixated in 10% buffered formalin for no more than 12 h, then treated in calcium chloride and plasma solution until clot formation.^20^, ^28^

According to the recommendations provided in the latest edition of the WHO Classification of Lung Tumors, a diagnosis of the histotype should be achieved in order to schedule the appropriate therapy, and a tumor sample should be preserved for prognostic and/or predictive purposes. In fact, IHC analysis should be limited to poorly differentiated NSCLC samples,^27^ in which thyroid transcription factor 1 (TTF‐1) and p40 expression could be helpful to determine adenocarcinoma or squamous cell carcinoma histotype, respectively. On the contrary, small cell features, necrosis and nuclear molding suggestive for SCLC hystotype could be confirmed by at least one neuroendocrine marker such as chromogranin, synaptophysin, and CD56 and a high proliferative index documented by immunoreactivity for the Mib‐1 antibody.^27^, ^37^

In order to maximize diagnostic, predictive and prognostic informations from EBUS‐TBNA samples, CB specimens stained with Hematoxylin and Eosin were submitted to an internal quality control group, formed by at least two pathologists, or a pathologist and a cytologist, in order to better handle the cytological samples, avoiding unnecessary IHC and/or limiting immunoprofiling of pulmonary neoplasm (primary vs. metastatic). Once NSCLC was confirmed as advanced, ALK, ROS1 and PD‐L1 expressions and any gene rearrangements by FISH or mRNA transcript analysis were performed.

Our study on 464 consecutive EBUS‐FNAs has documented 95.5% of adequacy for site and 92.6% of adequacy for diagnosis, allowing us to define the lung cancer histotype according to the WHO Classification of Lung Tumors, or to identify the primary site of pulmonary metastasis.

Also, a case of primary pulmonary Hodgkin's lymphoma has been diagnosed, as previously described, by an immunohistochemical analysis on CB.^20^, ^28^

In advanced NSCLC, the CAP/IASLC/AMP guidelines state that ALK genetic abnormalities can be IHC screened based on highly specific antibodies (D5F3 or 5A4 clones) on CB if cytological material is the only tumor sample available.^15^ In fact, in cases of equivocal ALK immunoreactivity, CB allows FISH assays to be performed, which are necessary to confirm ALK rearrangement.^31^ Moreover, ROS1 gene translocation can be supposed by IHC on CB using rabbit monoclonal antibody D4D6, but ROS1 positivity should always be followed by FISH or molecular analysis to confirm specific gene alteration, suitable on CB.^31^, ^37^

PD‐L1 testing is currently validated only on formalin‐fixed paraffin‐embedded materials (histological and cytological samples as clots or pellets) but not on cytological smears.^38^ In the literature, the TPS PD‐L1 concordance between smears and the respective surgical samples has been reported to be low.^38^ Consequently, a proportion of advanced NSCLC patients cannot be tested for PD‐L1 expression and could be precluded from first‐line single‐agent immune checkpoint inhibitor treatment if only smears are obtained. Conversely, a growing body of the literature is currently available on the performance of PD‐L1 IHC on CB.

Studies carried out on matched cytological and histological samples from the same patients have reported comparable results between CBs, surgical resections and small biopsy specimens in terms of adequacy rate, level of PD‐L1 expression, and clinical outcomes.^39^ However, it is very difficult to compare these studies because of the variety of the existing methods and workflows. Moreover, most studies about PD‐L1 assessment on cytological samples have provided only few details on laboratory processing and fixation time.^30^, ^40^

A very interesting recent study has demontrated that a lengthy fixation could detrimentally affect PD‐L1 IHC on CBs. More specifically, a prolonged fixation time of CBs did not significantly affect the performance of the SP263 assay, whereas 22C3 performance in terms of TPS and signal intensity was seriously influenced in a laboratory developed test (LDT).^39^

In our series, 83.6% of the NSCLC patients were found to be at an advanced stage needing prognostic and predictive factor assessment to select the most appropriate therapy; 96% of the adenocarcinoma samples were suitable for ALK, ROS1 and PD‐L1 assessment, whereas, mainly due to tumor necrosis and/or small amount of viable neoplastic cells, just 81% of the squamous cell carcinomas were adequate for TPS PD‐L1 assessment. All CBs related to carcinoma NOS were adequate for ALK, ROS1 and PD‐L1 assessment and also molecular profiling.

Finally, 93.8% of the all primary lung cancer samples (localized and advanced stages) were adequate for molecular profiling, and our yield appears to be better than that of the most recent large series of 74 CBs from NSCLC patients in which 90.6% of adequacy for molecular profiling was observed.^33^ In this study, no data on fixing procedure and timing were reported.^33^

In patients with metastatic disease, from both pulmonary or an extra‐thoracic primary, in the presence of clinical indication, molecular analysis was performed but description and discussion of the results of molecular profile are beyond the aims of our study.

Most interestingly, the median turnaround time (TAT) of diagnosis in our study was about 30 h, resulting in shorter hospitalization time for patients.

These encouraging results are explained as follows. First, the planned interventional pulmonologist evaluation of imaging of all suspected lung cancer patients the day before the EBUS procedure allowed us to accurately select the patients and site of sampling. Moreover, the internal agreement between clinicians and the Pathology Unit allowed us to handle the cytological samples in a controlled and timely manner. In March 2014, the CB method was optimized with the help of an expert cytology technician. Finally, our cytological internal quality control ensured an accurate diagnosis and better management of cytological samples, thus avoiding useless immunohistochemical staining and preservation of materials for molecular profiling.

However, our study has some limitations.

First, the number of lung lesion samples is very small because patients are only approached by interventional pulmonologists in our center when there is unique manifestation of the disease, instead preferring to sample the accompanying adenopathies to obtain staging as well as diagnosis of illness. For solitary pulmonary nodules they also practice transbronchial histological biopsies using dedicated forceps instead of EBUS needles and subsequently to the identification of the target obtained with radial ultrasound mini‐probes or fluoroscopy.

Second, some follow‐up data of patients with EBUS‐TBNA adequate for site but negative for neoplastic lesions and all radiological findings regarding size of mediastinal lymph nodes are lacking.

However, we believe that our monoinstitutional experience could be the first step toward a more in‐depth study on the utility of standardization for sampling, time‐fixation and handling of cytological samples with CB from EBUS‐TBNA specimens in NSCLC patients.

In conclusion, a correct diagnosis and subtyping of NSCLC samples is imperative to establish adequate therapy and the most appropriate clinical management. In advanced NSCLC patients, a less invasive procedure such as EBUS‐TBNA allows a more accurate acquisition of samples. A wise cytological smear management together with the handling and standardization of CB obtained from EBUS‐TBNA could represent an effective method to increase the adequacy of the tumor specimen for both diagnosis and molecular profiling. For this reason, an adequate handling of cytological samples and a scheduled workflow involving clinicians, technicians, pathologists and molecular pathologists is of paramount importance.

## AUTHOR CONTRIBUTIONS

**Conceptualization**: Paola Parente, Paolo Graziano; **Methodology**: Paola Parente, Cristiano Carbonelli, Giovanni Biancofiore, Andi Sukthi, Concetta Martina Di Micco, Matteo Vairo, Paolo Fuso, Paolo Graziano; **Validation**: Paola Parente, Cristiano Carbonelli, Marco Taurchini, Paolo Graziano; **Formal Analysis**: Paola Parente, Cristiano Carbonelli, Marco Taurchini, Paolo Graziano; **Investigation**: Paola Parente, Cristiano Carbonelli, Giovanni Biancofiore, Paolo Graziano; **Resources**: Paolo Graziano; **Data Curation**: Paola Parente, Cristiano Carbonelli, Giovanni Biancofiore, Andi Sukthi, Concetta Martina Di Micco, Matteo Vairo, Paolo Fuso, Paolo Graziano; **Writing – Original Draft Preparation**: Paola Parente, Cristiano Carbonelli, Paolo Graziano; **Writing – Review & Editing**: Paola Parente, Cristiano Carbonelli, Giovanni Biancofiore, Andi Sukthi, Concetta Martina Di Micco, Matteo Vairo, Paolo Fuso, Marco Taurchini, Paolo Graziano; **Visualization** Paola Parente, Cristiano Carbonelli, Paolo Graziano; **Supervision**: Paola Parente, Paolo Graziano; **Project Administration**: Paola Parente, Paolo Graziano.

## CONFLICT OF INTEREST

The authors have no conflicts of interest to declare.
